# First Detection of Tetrodotoxins in the Cotylean Flatworm *Prosthiostomum trilineatum*

**DOI:** 10.3390/md19010040

**Published:** 2021-01-18

**Authors:** Rei Suo, Maho Kashitani, Hikaru Oyama, Masaatsu Adachi, Ryota Nakahigashi, Ryo Sakakibara, Toshio Nishikawa, Haruo Sugita, Shiro Itoi

**Affiliations:** 1 Department of Marine Science and Resources, Nihon University, Fujisawa, Kanagawa 252-0880, Japan; monton.mk@gmail.com (M.K.); brhi20501@g.nihon-u.ac.jp (H.O.); sugita.haruo@nihon-u.ac.jp (H.S.); 2 Graduate School of Pharmaceutical Sciences, Tohoku University, Aoba, Aramaki, Aoba-ku, Sendai 980-8578, Japan; masaatsu.adachi.d7@tohoku.ac.jp; 3 Laboratory of Organic Chemistry, Graduate School of Bioagricultural Sciences, Nagoya University, Chikusa, Nagoya 464-8601, Japan; nakahiga.ry@gmail.com (R.N.); sakakibara.ryou@ma.mt-pharma.co.jp (R.S.); nisikawa@agr.nagoya-u.ac.jp (T.N.)

**Keywords:** tetrodotoxin, analog, LC-MS/MS, flatworms, Polycladida, Cotylea

## Abstract

Several polyclad flatworm species are known to contain high levels of tetrodotoxin (TTX), but currently TTX-bearing flatworms seem to be restricted to specific *Planocera* lineages belonging to the suborder Acotylea. During our ongoing study of flatworm toxins, high concentrations of TTXs were detected for the first time in the flatworm *Prosthiostomum trilineatum*, suborder Cotylea, from the coastal area of Hayama, Kanagawa, Japan. Toxin levels were investigated by high performance liquid chromatography-tandem mass spectrometry (LC-MS/MS), revealing that this species contains comparable concentrations of toxins as seen in planocerid flatworms such as *Planocera multitentaculata*. This finding indicated that there may be other species with significant levels of TTXs. The distribution of TTXs among other flatworm species is thus of great interest.

## 1. Introduction

Tetrodotoxin (TTX), a well-known potent neurotoxin first isolated from pufferfish [1,2,3], has been discovered in a wide range of marine and terrestrial organisms, such as amphibians, flatworms, crustaceans, mollusks, and several species of marine bacteria [4,5,6]. TTX and its analogs are grouped with sodium channel blockers, preventing sodium ion influx through sodium channels in an excitable membrane, resulting in serious toxicity [7,8,9]. The toxicity of TTX-bearing organisms depends upon geography and season. In addition, the wide distribution of TTX across phylogenetically diverse organisms has led to the hypothesis of a bacterial origin of TTX, either from accumulation through the food chain or from symbiotic relationships with TTX-bearing organisms [5,10,11,12]. However, the origin of TTX remains controversial, and the biosynthetic pathway remains unclear [13,14].

Some marine polyclad flatworms are known to contain high concentrations of TTX and are thought to be one of the animals that might be responsible for the presence of TTX in some marine organisms. The sea slug *Pleurobranchaea maculata*, the organisms ascribable to the neurotoxicosis of dogs in some beaches of Auckland, New Zealand, are reportedly toxificated by feeding on the TTX-bearing flatworm *Stylochoplana* sp. [15,16,17]. Additionally, we have investigated the ecological function of *Planocera multitentaculata,* inhabiting the coastal area of Hayama, Kanagawa, Japan and have confirmed the following: the DNA sequence of the flatworm *Pl. multitentaculata* was detected in the intestinal contents of juveniles and young of the pufferfish *Takifugu niphobles* (at present *Takifugu alboplumbeus*); the pufferfish at various growth stages effectively toxified itself by feeding on the flatworm *Pl. multitentaculata* and its eggs; and, non-toxic pufferfish became toxified after feeding on the flatworm *Pl. multitentaculata* [18,19]. Moreover, Next Generation Sequencing (NGS) analysis of the cytochrome *c* oxidase subunit I (COI) gene of the intestinal contents from juveniles of TTX-bearing organisms demonstrated that the majority of the sequence is identical to that of *Pl. multitentaculata* [20]. These results indicate that the flatworm contributes to the toxification of pufferfish at various life stages and functions as one of the major TTX sources for other TTX-bearing fish species. This leads to great interest in finding out the distribution of TTXs among other flatworm species.

The polyclad flatworms have been traditionally divided into two suborders, Cotylea and Acotylea, which are distinguished by the presence or absence of a ventral sucker behind the genital openings [21,22,23]. They are globally distributed in the marine environment, found under coastal rocks and in interstitial spaces. Currently, the toxins have been detected within the suborder Acotylea, such as *Pl. multitentaculata*, *Planocera reticulata* and *Stylochoplana* sp., but toxification by TTX is still undetected within the suborder Cotylea [16,17,24,25,26,27]. However, our ongoing research on toxins from marine polyclad flatworms has now detected a high concentration of TTX and its analog from the cotylean flatworm, *Prosthiostomum trilineatum,* found in a coastal area of Hayama, Kanagawa, Japan. Herein, we report the first detection of TTXs within this suborder and discuss its habitat, relationship with other marine organisms and potential adverse human health risk.

## 2. Results and Discussion

### 2.1. External Morphology and Molecular Phylogenetic Inference of the Flatworm Pr. trilineatum

**Figure 1:**
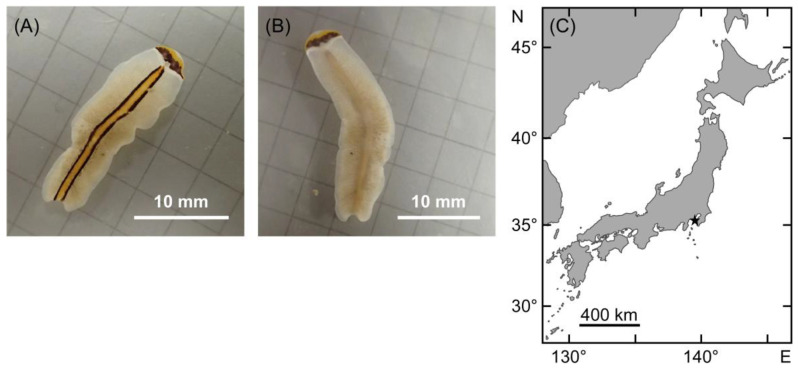
External morphology of *Prosthiostomum trilineatum* collected in this study. (**A**,**B**) represent dorsal and ventral views of specimen 1, respectively. (**C**) represents map of sampling location (marked by a star).

Two flatworm specimens were collected from the coastal area of Hayama, Kanagawa, Japan under stones in the intertidal zone, and a rocky bottom at a depth of approximately 10 m. These were identified as *Pr. trilineatum* based on the description of external morphology [28,29]; broadly elongated body, rounded anteriorly and tapered posteriorly; longitudinal orange-yellow stripe bordered by black band running medially (Figure 1).

**Figure 2:**
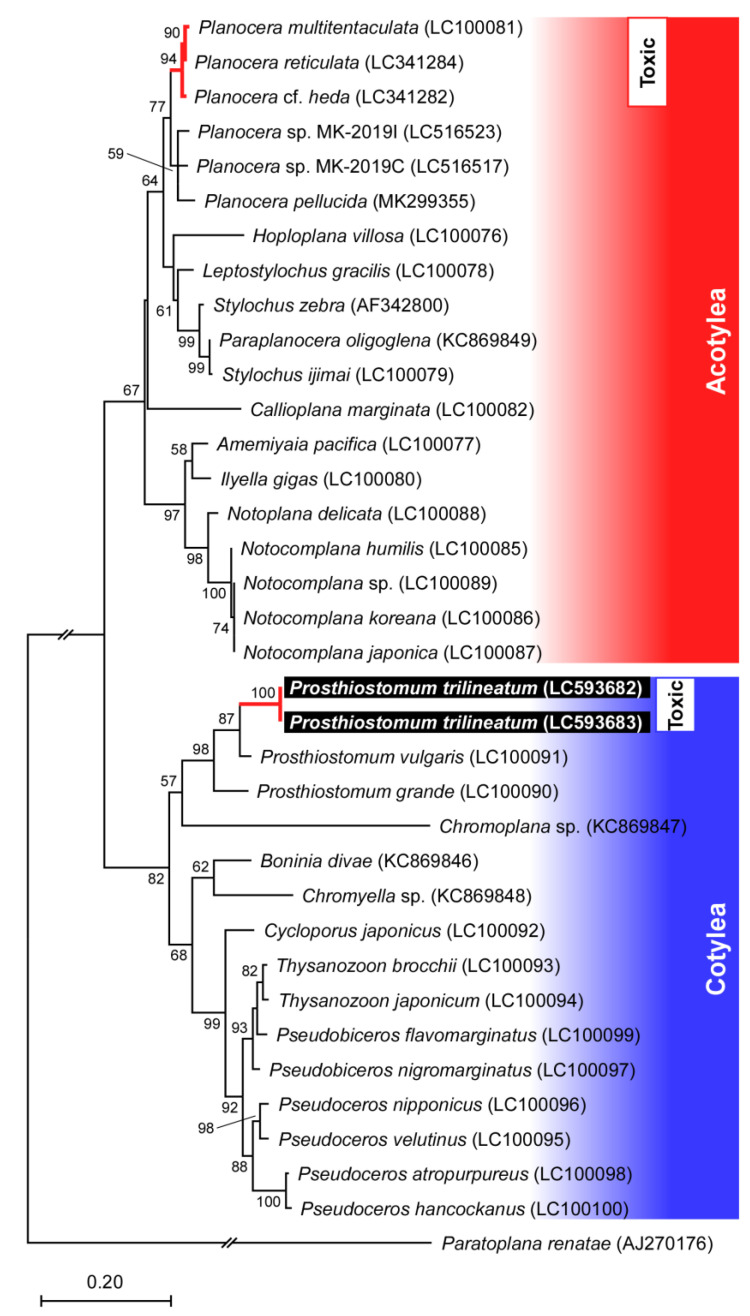
Phylogenetic position of *Prosthiostomum trilineatum* among polyclad species inferred from the 28S rRNA gene sequence. The phylogenetic tree was constructed by maximum likelihood analysis. Numbers at branches denote the bootstrap percentages from 1000 replicates. The accession numbers for the sequences are shown in parentheses. The accession numbers LC593682 and LC593683 refer to those deposited in the DDBJ/EMBL/GenBank databases in this study. The sequence from *Paratoplana renatae* was used as the outgroup. Only bootstrap values exceeding 50% are presented. The scale refers to nucleotide substitutions per site.

Additionally, partial sequences of the 28S rRNA (approximately 1100 bp) were obtained from both specimens, and the resulting sequences showed high similarity with those of related cotylean flatworm species, *Prosthiostomum vulgaris* for 94.4% and *Prosthiostomum siphunculus* for 92.4%. Maximum likelihood analysis based on 28S rRNA sequences from various polyclad flatworms revealed that the *Pr. trilineatum* specimens in this study formed a cluster containing sequences from other *Prosthiostomum* species in the suborder Cotylea (Figure 2). The DNA sequences of the 28S rRNA gene fragment from this study have been submitted to the DDBJ/EMBL/GenBank databases.

### 2.2. TTXs Analysis

**Figure 3:**
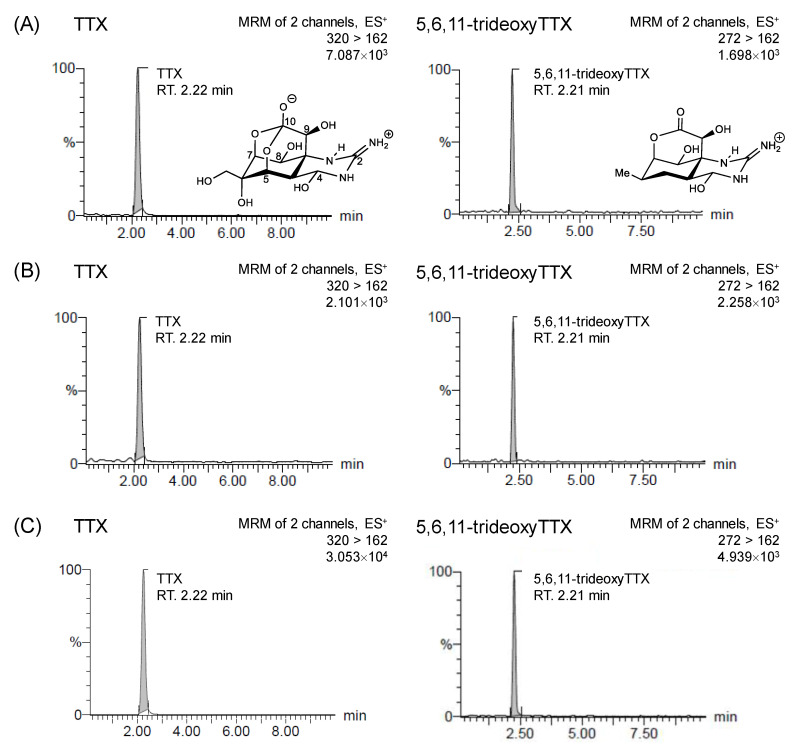
Liquid chromatography-tandem mass spectrometry (LC-MS/MS) chromatograms with multiple reaction monitoring mode (TTX: *m*/*z* 320 > 162, 5,6,11-trideoxyTTX: *m*/*z* 272 > 162). (**A**) Standards of TTXs, (**B**) the extracts from *Prosthiostomum trilineatum* specimen 1, (**C**) the extracts from *Pr. trilineatum* specimen 2. The concentration of the standard is shown as below: TTX 50 ng/mL, 5,6,11-trideoxyTTX 50 ng/mL.

It is well-known that the TTX-bearing organisms, such as pufferfish and newt, contain 4-*epi*TTX and 4,9-anhydroTTX, which are the chemically equilibrium analogs of TTX [30]. Additionally, several non-equilibrium TTX analogs with different oxidation states and stereochemistries have been isolated [31,32,33,34,35,36,37,38]. In our study, we focused on the detection of the major components, TTX and its analog 5,6,11-trideoxyTTX. The compound mixture containing TTX and 5,6,11-trideoxyTTX was used as the standard, and detected at *m*/*z* 320 > 302 for qualification and *m*/*z* 320 > 162 for quantification of TTX, and at *m*/*z* 272 > 254 for qualification and *m*/*z* 272 > 162 for quantification of 5,6,11-trideoxyTTX. The amount of TTXs in the two individual flatworms was calculated by measuring extracts with appropriate dilution, respectively. High concentrations of TTX and 5,6,11-trideoxyTTX were detected from both extracts of the specimens. The total amount of TTX in the body of specimen 1 and 2 was calculated at 223 μg/individual and 8.4 μg/individual, respectively, whereas 5,6,11-trideoxyTTX was 170 μg/individual and 6.2 μg/individual, respectively (Figure 3, Table 1).

So far, TTXs have been detected from the acotylean flatworms *Pl. multitentaculata*, *Pl. reticulata, Stylochoplana* sp., and *Planocera* sp. in significant levels [16,17,24,25,26,27]. Previously, we have investigated the toxicity of the acotylean flatworms *Pl. multitentaculata* (*n* = 122) and *Pl. reticulata* (*n* = 21), and found that all individuals contained TTXs [39,40,41]. Based on the phylogenetic tree of polyclad flatworms and toxin detection data as reported so far, TTX-bearing flatworm species seem to be restricted to specific *Planocera* lineages in the suborder Acotylea [41] (Figure 2). However, the data present in this study raise the intriguing possibility that the distribution of TTXs among marine polyclad flatworms might be more diverse than expected. Although TTX contents vary between the individuals, and the number of samples (*n* = 2) in this study is limited, both *Pr. trilineatum* specimens contained levels of TTXs comparable to those of other planocerid flatworms [26,39,40]. As far as we know, in the last 10 years, the cotylean flatworm *Pr. trilineatum* has rarely been observed in the intertidal zone of Hayama. Thus, in this study, only two individuals were subjected to the TTXs analysis due to the difficulty of sample collection. Further study will be required to confirm whether the toxins are constantly or variably present in *Pr. trilineatum*. Besides, a similar restricted taxonomic distribution of TTX might also be seen in the *Prosthiostomum* and related lineages; therefore, a wider and more intensive search of toxins distribution among cotylean flatworms, especially among the genus *Prosthiostomum*, is necessary.

### 2.3. Prosthiostomum trilineatum and Possible Relationship with Other Marine Organisms

The distribution of toxins among individual *Pr. trilineatum* or its presence at specific geographical areas have not been investigated. The distribution of *Pr. trilineatum* is quite wide, extending from the North Equatorial Current to the Kuroshio Current including Japan, Micronesia, Guam, and Papua New Guinea, and even to Singapore and India [28,29,42,43], suggesting that the sampling site for this study may be the northern limit in the Kuroshio basin. If so, *Pr. trilineatum* may be more abundant at the headwaters of the Kuroshio Current. Based on our study and the other reports regarding the collection of *Pr*. *trilineatum*, we deduce that *Pr*. *trilineatum* probably inhabits the deeper water (≥10 m), which is deeper than our collection sites. However, the biology and ecology of *Pr. trilineatum* are unstudied, and neither the spawning season nor the amount of biomass around Hayama are clear. Further studies to identify *Pr. trilineatum* habitat and toxin distribution are needed.

In the current study, flatworms were collected from the coastal area of Hayama, close to the geographical distribution of *Pl. multitentaculata* and *Pl. reticulata*. Our previous report showed that the pufferfish *T. alboplumbeus* inhabiting an intertidal zone of Hayama effectively acquired the toxins by feeding on *Pl. multitentaculata*, and its eggs and planktonic larvae [19,44]. Although the biomass of *Pr. trilineatum* seems to be limited compared with that of *Pl. multitentaculata* in the waters around Hayama area, there is a possibility that *Pr. trilineatum* also contributes to the toxification of pufferfish.

Generally, flatworms are considered carnivores that feed on immobile animals such as ascidians, mollusks, and bryozoans [45]. The origin of TTX in pufferfish, as well as in flatworms, is still unresolved, although TTX is hypothesized to be biosynthesized by bacteria and bio-accumulated through the food chain [5,12]. Therefore, it is important to investigate how the flatworms acquired the toxins, and why they possess highly concentrated toxins. In addition, it is necessary to investigate the effect of flatworms on the toxification of edible marine organisms, which are traditionally thought of as non-toxic. Recently, there has been a problematic world-wide increase in TTXs detection in edible fish, bivalves, and gastropods. TTXs have spread rapidly in the Mediterranean with levels high enough to cause food poisoning [46,47,48]. Additionally, TTXs have been detected in high concentrations in over ten marine bivalve and gastropod species in the Pacific and Mediterranean [49,50,51,52,53,54]. However, many aspects regarding the origin of TTX in shellfish as well as pufferfish are unclear, accelerating the interest surrounding TTX in these organisms. In our previous research, we detected high percentages of the toxic planocerid DNA sequence in the gut content of TTX-bearing fishes, and we suggested that planocerid flatworm larvae are involved in toxification process of TTX-bearing marine fishes around the Sakishima Islands, and Hayama, Kanagawa, Japan, [19,20]. Although there is no direct evidence to show that flatworms are the origin of toxified edible marine organisms, more studies, such as information about the relationship between feeding habits of TTX-bearing organisms and flatworms or other planktonic organisms, will provide evidence on the origin of TTX in marine habitats. This will ultimately lead to better protection of human health.

## 3. Materials and Methods

### 3.1. Flatworm Specimens

Flatworms were collected under stones in the intertidal zone (specimen 1), and a rocky bottom at a depth of approximately 10 m (specimen 2) from the coastal area of Hayama, Kanagawa, Japan (35°15′ N, 139°34′ E). The mass of the specimens could not be recorded due to their very small size. Each individual was photographed and then stored at −20 °C until DNA analysis and TTX extraction.

### 3.2. Preparation of Sample Solutions of the Flatworm for LC-MS Analysis

The flatworms after a small portion was excised were subjected to TTXs extraction in 1 mL of 0.1% acetic acid by heating for 10 min in boiling water. The extracts were centrifuged for 20 min at 14,000× *g*; then the supernatants were filtered through a membrane of pore size 0.45 μm (SupraPure Syringe Filter, PTFE-Hydrophilic, Recenttec, Taipei, Taiwan) and subjected to liquid chromatography-tandem mass spectrometry (LC-MS/MS) analysis.

### 3.3. LC-MS/MS Analysis

LC-MS/MS with multiple reaction monitoring (MRM) mode was performed using a Quattro Premier XE (Waters, Milford, MA, USA) with an electrospray ionization (ESI) source coupled to an Acquity UPLC system (Waters). The sample solutions were analyzed by LC-MS/MS at a flow rate of 0.3 mL/min on an Atlantis HILIC Silica column (2.1 mm × 150 mm, 5 μm, Waters) at 40 °C. Gradient elution was performed using solvent A (H_2_O containing 0.1% formic acid) and solvent B (MeCN) with the following linear gradient combination: 95% (B), decreasing to 40% (B) after 0.1 min, then kept for 7.9 min, and back to 95% (B), and finally kept at 95% (B) for 2.0 min before the next injection [15]. In advance of sample analysis, the standard calibration curve was created using 1 to 100 ng/mL of TTXs standards (TTX and 5,6,11-trideoxyTTX [55]) which showed good linearity and precision (TTX: *y* = 83.6085*x* + 708.342, *R*^2^ = 0.96; 5,6,11-trideoxyTTX: *y* = 18.8439*x* − 2.37614, *R*^2^ = 0.99). The optimized transitions for TTX were *m*/*z* 320 > 302 for confirmation and *m*/*z* 320 > 162 for quantification, and those for 5,6,11-trideoxyTTX were *m*/*z* 272 > 254 for confirmation and *m*/*z* 272 > 162 for quantification, as shown below (Table 2).

### 3.4. Molecular Analysis

Total genomic DNA was extracted from the excised tissue using standard phenol–chloroform extraction with some modification [56]. The 28S rRNA genes were PCR-amplified from the extracted DNA using the primers, HRNT-F2 (5′-AGTTC AAGAG TACGT GAAAC C-3′) and HRNT-R2 (5′-AACAC CTTTT GTGGT ATCTG ATGA-3′) [56]. The PCR reaction mixture contained 1 μL of DNA template, 2 μL of 10 × buffer for Ex Taq, 1.6 μL of dNTPs, 2.6 μL of 5 mM of each primer, 0.625 unit of Ex Taq (Takara Bio, Shiga, Japan), and H_2_O for a total volume of 20 μL. The PCR reaction was performed on a T100 Thermal Cycler (Bio-Rad) as follows: initial denaturation at 95 °C for 1 min, 35 cycles of denaturation 95 °C for 10 s, annealing at 55 °C for 30 s, and a final elongation for 1 min at 72 °C. The PCR products were electrophoresed on 2% agarose gel in 1 × TAE buffer, stained with ethidium bromide, and purified followed by direct sequencing. The resulting strands were sequenced with a 3130*xl* genetic analyzer (Applied Biosystems, Foster City, CA, USA) using a BigDye Terminator v3.1 Cycle Sequencing Ready Reaction Kit (Applied Biosystems). The sequences of the 28S rRNA gene were submitted to the DDBJ/EMBL/GenBank databases under the accession number LC593682-LC593683. Then, 28S rRNA gene sequences for the following species were obtained from the DDBJ/EMBL/GenBank databases: *Stylochus zebra* (AF342800), *Boninia divae* (KC869846), *Chromoplana* sp. (KC869847), *Chromyella* sp. (KC869848), *Paraplanocera oligoglena* (KC869849), *Planocera pellucida* (MK299355), *Amemiyaia pacifica* (LC100077), *Leptostylochus gracilis* (LC100078), *Stylochus ijimai* (LC100079), *Discoplana gigas* (LC100080), *Pl. multitentaculata* (LC100081), *Callioplana marginata* (LC100082), *Notocomplana humilis* (LC100085), *Notocomplana koreana* (LC100086), *Notocomplana japonica* (LC100087), *Notoplana delicata* (LC100088), *Notocomplana* sp. (LC100089), *Prosthiostomum grande* (LC100090), *Pr. vulgaris* (LC100091), *Cycloporus japonicus* (LC100092), *Thysanozoon brocchii* (LC100093), *Thysanozoon japonicum* (LC100094), *Pseudoceros velutinus* (LC100095), *Pseudoceros nipponicus* (LC100096), *Pseudobiceros nigromarginatus* (LC100097), *Pseudoceros atropurpureus* (LC100098), *Pseudobiceros flavomarginatus* (LC100099), *Pseudoceros hancockanus* (LC100100), *Planocera* cf. *heda* (LC341282), *Pl. reticulata* (LC341284), *Planocera* sp. MK-2019C (LC516517), *Planocera* sp. MK-2019I (LC516523), and *Paratoplana renatae* (AJ270176). The 28S rRNA gene sequences of acotylean and cotylean flatworms were aligned using Clustal W [57]. A phylogenetic analysis based on the partial sequence of 28S rRNA gene (approximately 700 bp) was constructed by the maximum likelihood method with Kimura 2-parameter + G with 1000 bootstrap using MEGA X version 10.1 [58].

## 4. Conclusions

In summary, we report the first detection of TTX and 5,6,11-trideoxyTTX from the cotylean flatworm, *Pr. trilineatum,* collected from the coastal area of Hayama, Kanagawa, Japan. Although investigation of further specimens is suggested, our results suggest that the distribution of TTXs among flatworms might be wider than speculated, and it would thus be interesting to search for TTX or its precursor analogs in other flatworms. These findings will be useful in determining the origin of TTX, and elucidating its biosynthetic pathway.

## Figures and Tables

**Table 1:** The contents of TTXs in *Pr. trilineatum*.

Compounds	Content (μg/individual)	
	Specimen 1	Specimen 2
TTX	223	8.4
5,6,11-trideoxyTTX	170	6.2

**Table 2:** MRM transitions used for detection and quantification of TTXs.

Compounds	Transition	Cone (V)	CE (V)
TTX	320 > 302	42	24
	320 > 162	42	38
5,6,11-trideoxyTTX	272 > 254	46	19
	272 > 162	46	28

## Data Availability

Data is contained within the article.
